# Reduction in Subventricular Zone-Derived Olfactory Bulb Neurogenesis in a Rat Model of Huntington’s Disease Is Accompanied by Striatal Invasion of Neuroblasts

**DOI:** 10.1371/journal.pone.0116069

**Published:** 2015-02-26

**Authors:** Mahesh Kandasamy, Michael Rosskopf, Katrin Wagner, Barbara Klein, Sebastien Couillard-Despres, Herbert A. Reitsamer, Michael Stephan, Huu Phuc Nguyen, Olaf Riess, Ulrich Bogdahn, Jürgen Winkler, Stephan von Hörsten, Ludwig Aigner

**Affiliations:** 1 Institute of Molecular Regenerative Medicine, Paracelsus Medical University, Salzburg, Austria; 2 Spinal Cord Injury and Tissue Regeneration Center Salzburg, Paracelsus Medical University, Salzburg, Austria; 3 Department of Neurology, University Hospital Regensburg, Regensburg, Germany; 4 Institute of Experimental Neuroregeneration, Paracelsus Medical University, Salzburg, Austria; 5 Department of Ophthalmology, SALK, Paracelsus Medical University, Salzburg, Austria; 6 Institute of Functional and Applied Anatomy, Hannover Medical School, Hannover, Germany; 7 Department of Medical Genetics, University of Tübingen, Tübingen, Germany; 8 Division of Molecular Neurology, Friedrich-Alexander-University Erlangen-Nürnberg, Erlangen, Germany; 9 Experimental Therapy, Friedrich-Alexander-University Erlangen-Nurnberg, Erlangen, Germany; University of Oxford, UNITED KINGDOM

## Abstract

Huntington’s disease (HD) is an inherited progressive neurodegenerative disorder caused by an expanded CAG repeat in exon 1 of the huntingtin gene (HTT). The primary neuropathology of HD has been attributed to the preferential degeneration of medium spiny neurons (MSN) in the striatum. Reports on striatal neurogenesis have been a subject of debate; nevertheless, it should be considered as an endogenous attempt to repair the brain. The subventricular zone (SVZ) might offer a close-by region to supply the degenerated striatum with new cells. Previously, we have demonstrated that R6/2 mice, a widely used preclinical model representing an early onset HD, showed reduced olfactory bulb (OB) neurogenesis but induced striatal migration of neuroblasts without affecting the proliferation of neural progenitor cell (NPCs) in the SVZ. The present study revisits these findings, using a clinically more relevant transgenic rat model of late onset HD (tgHD rats) carrying the human HTT gene with 51 CAG repeats and mimicking many of the neuropathological features of HD seen in patients. We demonstrate that cell proliferation is reduced in the SVZ and OB of tgHD rats compared to WT rats. In the OB of tgHD rats, although cell survival was reduced, the frequency of neuronal differentiation was not altered in the granule cell layer (GCL) compared to the WT rats. However, an increased frequency of dopamenergic neuronal differentiation was noticed in the glomerular layer (GLOM) of tgHD rats. Besides this, we observed a selective proliferation of neuroblasts in the adjacent striatum of tgHD rats. There was no evidence for neuronal maturation and survival of these striatal neuroblasts. Therefore, the functional role of these invading neuroblasts still needs to be determined, but they might offer an endogenous alternative for stem or neuronal cell transplantation strategies.

## Introduction

Huntington’s disease (HD) is an autosomal dominant genetic neurodegenerative disorder of the basal ganglia [1]. HD is caused by the expansion of >39 CAG triplet segments in the exon 1 of the Huntingtin gene (HTT) encoding the Huntingtin protein (Htt) [2,3]. The polyglutamine (PolyQ) repeat length in the HTT gene is proposed to influence age of onset of the disease with symptoms that include movement, cognitive and psychiatric disorders [4,5]. The main pathological feature of the disease is progressive degeneration of medium spiny neurons (MSN) leading to marked atrophy of the striatum [1,6]. The roles of normal Htt protein as well as the neuropathogenesis induced by the mutant Htt protein are yet to be determined. Currently, there is no effective treatment available for HD. Hence, further investigations of the neuropathogenesis and endogenous regenerative potential of the HD brain are needed with the ultimate aim to develop innovative strategies to treat this devastating disease.

The subventricular zone (SVZ) of the lateral ventricle wall is a major source of multipotent neural stem and progenitor cells (NSC and NPCs) within the adult brain [7,8,9]. NPCs derived immature neurons or neuroblasts in the SVZ are capable of long-distance migration along the rostral migratory stream (RMS), where they differentiate into functional neurons in the olfactory bulb (OB) [7,10,11,12]. Previously, the striatum has been considered as non-neurogenic area of the adult brain [13], but a number of studies have suggested the production of new neurons in the striatum of nonhuman primates [14], rats [15], and rabbits [16]. Notably, the turnover of a portion of newborn neurons has also been reported recently in the striatum of adult human brains, whereas in HD patients, the striatum is apparently depleted of newly generated neurons [17].

The SVZ response to brain injuries [18,19,20,21] and neurodegeneration [22,23,24] has increasingly been recognized as a potential mode for self-regeneration in the adult brain. Pathogenic stimuli provoked by stroke, epilepsy or neurodegeneration (including HD) influence the proliferation of NPCs in the SVZ and attract their progeny towards the degenerating area, i.e. is the striatum in the case of HD [19,23,25]. Hence, it can be considered that ectopic migration of a subset of endogenous neuroblasts from the SVZ redirected towards the injured brain regions could partially support self-repair mechanisms of the brain leading to functional recovery.

Studies on post-mortem human brain tissues have revealed that cell proliferation is increased in the sub-ependymal layer (SEL) of HD brains [22], i.e. the region that corresponds to the SVZ in rodents. Though this study identifies the proliferating cells as beta-III Tubulin or as GFAP immunoreactive cells, it did not provide evidence for the proliferating cells being neural stem or progenitors. The increased proliferation of NPCs in the SVZ, together with neuroblasts migrating towards the acutely lesioned striatum, is also observed in a toxic rat model of HD, in which HD symptoms are caused by injection of quinolinic acid (QA) [23]. In our earlier studies, we have reported that though there was no difference in the proliferation of NPCs in the SVZ of R6/2 mice [26] carrying a human HD gene with ~150 CAG repeats [27], the migration of doublecortin (DCX) expressing neuroblasts into the affected striatum was evident. Further, we also noticed that these ectopically migrating neuroblasts were not expressing the mutant Htt protein, at least there were no detectable Htt aggregates. Nevertheless, they mostly failed to survive in the affected striatum of R6/2 mice [26]. Thus, the potential regenerative mechanism provoked due to the degeneration of medium spiny neurons (MSNs) in the HD brain is apparently not sufficient to provide a full striatal repair [17,26].

To better understand the endogenous neuronal recruitment into the HD striatum, we employed a transgenic rat model of HD (tgHD) carrying a human HTT gene with 51 CAG repeats under the control of the native rat HTT promoter [28] with a special focus on the SVZ-OB neurogenesis. This rat model recapitulates many of the pathological features of HD and reflects the human situation more closely compared to the R6/2 mouse model of HD [27,28].

## Materials and Methods

### Animals

Twelve months old male rats (WT = 8, tgHD = 16) were obtained from a colony of tgHD and WT littermates maintained at the central animal facility at the University of Hannover, Germany [28]. The presence of the HTT transgene in tgHD rats was confirmed by tail-DNA genotyping at the age of 3 weeks as previously described [28,29]. All experiments were carried out in accordance with the European Communities Council Directive of 24 November 1986 (86/609/EEC) and were approved by the local governmental commission for animal health, the “Regierung Mittelfranken” (file number: 54–2532.1–16/08).

### BrdU labelling

Dividing cells were labelled by intraperitoneal injection of the thymidine analogue BrdU (5-bromo-2-deoxyuridine) (Sigma, Steinheim, Germany) at 50 mg/kg of body weight (10 mg/ml of BrdU dissolved in a 0.9% (w/v) NaCl solution). For the cell proliferation analysis, rats were given two BrdU injections with an interval of 12 hours and sacrificed 24 hours post injection (WT = 4, tgHD = 8). To determine cell survival, BrdU injections were given daily for 5 consecutive days (day 1 to 5) and rats were sacrificed at day 30 (WT = 4, tgHD = 8).

### Tissue processing and immunostaining

Rats were deeply anesthetized using a mixture of ketamine (20.38 mg/ml), xylazine (5.38 mg/ml) and acepromazine (0.29 mg/ml) and perfused transcardially with 0.9% (w/v) NaCl solution followed by 4% paraformaldehyde in 0.1 M sodium phosphate solution (pH 7.4). Brains were dissected and post-fixed in paraformaldehyde overnight at 4°C. Tissue was then cryoprotected in 30% (w/v) sucrose in 0.1 M sodium phosphate solution (pH 7.4). Brains were cut into 40 μm sagittal sections using a sliding microtome on dry ice. Sections were stored at -20°C in cryoprotectant solution (ethylene glycol, glycerol, 0.1 M phosphate buffer pH 7.4, 1:1:2 by volume). Free-floating sections were treated with 0.6% H_2_O_2_ in Tris-buffered saline (TBS: 0.15 M NaCl, 0.1 M Tris–HCl, pH 7.5) for 30 min. For immunohistological detection of the incorporated BrdU, pre-treatment of tissues was performed as described previously [29]. Following extensive washes in TBS, sections were blocked with a solution composed of TBS, 0.1% Triton X 100, 1% bovine serum albumin (BSA) and 0.2% teleostean gelatine (Sigma, Taufkirchen, Germany) for 1h. The same buffer was also used during the incubation with antibodies. Tissues were incubated with primary antibodies for overnight at 4°C. For chromogenic immunodetection, sections were washed extensively and further incubated with biotin-conjugated species-specific secondary antibodies followed by a peroxidase–avidin complex solution from the Vectastain Elite ABC kit (Vector Laboratories, Burlingame, USA). The peroxidase activity of immune complexes was revealed with a solution of TBS containing 0.25 mg/ml 3, 3′-diaminobenzidine (DAB) (Vector Laboratories, Burlingame, USA), 0.01% (v/v) H_2_O_2_, and 0.04% (w/v) NiCl_2_. Sections were arranged on Superfrost Plus slides (Menzel, Braunschweig, Germany) and mounted in Neo-Mount (Merck, Darmstadt, Germany). For epifluorescence immunodetection, sections were washed extensively and incubated with fluorochrome-conjugated species-specific secondary antibodies for overnight at 4°C. Sections were put on slides and mounted in Prolong Antifade kit (Molecular Probes, Eugene, USA). Photo-documentation was done using a Leica microscope (Leica, Wetzlar, Germany) equipped with a Spot digital camera (Diagnostic Instrument Inc, Sterling Heights, USA) and epifluorescence observation was performed on a confocal scanning laser microscope (Leica TCS-NT, Wetzlar, Germany).

The following antibodies and final dilutions were used. Primary antibodies: rat anti-BrdU 1:500 (Oxford Biotechnology, Oxford, UK), goat anti-DCX (Doublecortin) (C-18) 1:500 (Santa Cruz Labs, Santa Cruz, USA), rabbit anti-phospho Smad 2 (1:100), mouse anti-Smad 2 (1:100) (Cell Signaling, USA), mouse anti-NeuN (neuronal nuclei) 1:500 (Chemicon, Temecula, USA) and sheep anti-tyrosine hydroxylase (TH; 1:500) (Chemicon, Temecula, USA). The secondary antibodies: donkey anti-goat,-mouse,-rabbit,-sheep or-rat conjugated with Alexa 488 (1:1000, Molecular Probes, Eugene, USA), rhodamine X (Dianova, Hamburg, Germany) or biotin (1:500; Jackson Immuno Research, West Grove, USA).

### Counting procedures

All analyses were performed on blind-coded slides. Every sixth section (240-μm interval) of one hemisphere was selected from each animal and processed for immunohistochemistry. BrdU-immunopositive cells in the SVZ, striatum and OB, were counted in every sixth section (240-μm intervals) from cell proliferation experiments and cell survival experiments, respectively. To investigate neurogenesis, the number of DCX positive cells in every twelfth section (480-μm interval) was determined. All cells that were stained by BrdU or DCX antibodies were counted with 400 x magnification on a light microscope (Leica, Wetzlar, Germany) and multiplied by 6 or 12 to obtain an estimate of the total immunopositive cell numbers. The reference volume was determined by tracing the SVZ, striatum as well as GCL and GLOM of the OB using a semi-automatic stereology system (Stereoinvestigator, MicroBrightField, Colchester, USA) as previously described in R6/2 mice[26]. All extrapolations were calculated for one hemisphere and should be doubled to represent the total brain.

To estimate the frequency of neuroblast generation from the pool of proliferating NPCs, a series of every sixth brain section (240-μm interval) from the proliferation paradigm was stained and analyzed for BrdU/DCX. To determine the frequency of neuronal differentiation, a series of every sixth brain section (240-μm interval) from the cell survival experiment was stained and analyzed for BrdU/NeuN or NeuN/TH double-stainings using a Leica TCS-NT confocal laser microscope (Leica Microsystems, Bensheim, Germany) equipped with a 40 x PL APO oil objective (1.25 numeric aperture) and a pinhole setting that corresponded to a focal plane of 2 μm or less.

### NPC cultures and migration assay

Neurosphere cultures of NPCs from adult hippocampus or SVZ were obtained as described previously[30]. Briefly, two to three months old female Fischer-344 rats (Charles River Deutschland GmbH, Germany) were decapitated. Tissue was aseptically removed and dissociated. Cells were resuspended in Neurobasal (NB) medium (Gibco BRL, Germany) supplemented with B27 (Gibco BRL, Germany), 2 mM L-glutamine (PAN, Germany), 100 U/mL penicillin / 0.1 mg/l streptomycin (PAN, Germany), hereafter referred to as NB/B27. For maintenance and expansion of the cultures, the NB/B27 was further supplemented with 2 μg/mL heparin (Sigma, Germany), 20 ng/mL FGF-2 (R&D Systems, Germany) and 20 ng/mL EGF (R&D Systems, Germany) (NB-A). Cultures were maintained in T-25 culture flasks at 37°C in a humidified incubator with 5% CO2. Neurosphere cultures from passage number 4 to 6 were used throughout this study and termed NPCs. For passaging these cells, the culture medium containing floating neurospheres was collected in a 15-mL Falcon tube and centrifuged at 120 x g for 5 minutes. The pellet was resuspended in 200 μL of 1% Accutase (PAA, Pasching, Austria) and triturated approximately 10 times using a pipette. Dissociated cells were centrifuged at 120 x g for 5 minutes, resuspended and reseeded.

For migration assays, 1.75 x 10^6^ cells were seeded into 800 μL NB-A for 12 hours. Thereafter (day1), cells were split into two T75 flasks and treated with 10 ng/mL TGF-beta1 or vehicle solution (4 mM HCL, 1mg/mL BSA) for 7 days (10 ng/mL TGF-beta1 or vehicle was supplied on day 1, 4, and 7). On day 8, TGF-beta1 or vehicle treated cells were seeded on poly-L-ornithine and laminin-coated coverslips in 24-well plates. Each well was further filled with 1 mL of NB all medium containing 10 ng/mL TGF-beta1 or vehicle solution respectively. Six wells were prepared for each condition, of which cell migration was analyzed in three wells. Micrographs were taken 18 hours post-seeding and the number of cells that migrated out per neurosphere was determined (Control = 3, TGF-beta1 = 3). The mean value of TGF-beta1 treatment was normalized with vehicle control (Control = 100%).

### Statistical analysis

The data are presented as mean values ± SD. The statistical analyses were carried out with Prism (Prism GraphPad Software, San Diego, CA). Student *t*-tests were used for analyzing the total numbers of BrdU-positive cells, the total numbers of DCX-positive cells, the percentage of BrdU/NeuN double-positive cells and migration assays. One-way analysis of variance (ANOVA) was used for the percentage of BrdU/DCX-positive cells, and a Tukey test comparison was performed for post hoc analysis. Statistical significance was defined as P<0.05.

## Results

### Reduced proliferation of NPCs in the SVZ of tgHD rats

**Fig 1:**
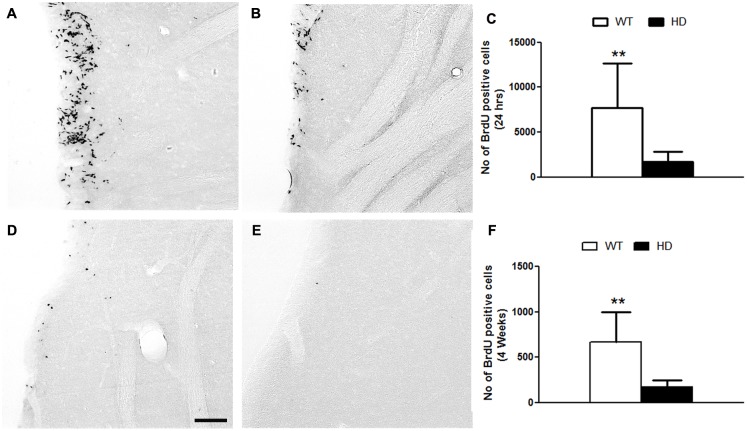
A, B) BrdU positive cells (24) in the SVZ of A) WT and B) tgHD rats. C) Quantitative analysis of BrdU positive cells after 24 hours treatment. Note, there is a significant reduction in proliferating cells in the SVZ of tg HD rats compared to WT(WT = 4, HD = 8; Student t test, P value = 0.0070). D-F) BrdU positive cells (30 days) in the SVZ of D) WT and E) tgHD rats. F) Quantitative analysis of BrdU positive cells (30 days). Note, there is a reduction in label retention in the SVZ of tgHD rats compared to WT (WT = 4, HD = 8; Student t test, P value = 0.0017). Sale bar = 100 μm.

To investigate neurogenesis in the SVZ-OB system of adult (12 months old) tgHD rats, we first analyzed cell proliferation in the SVZ by quantitative analysis of cells that were labelled with BrdU 24h before sacrificing the animals. As a result, the total number of BrdU-positive cells in the SVZ was significantly reduced in the SVZ of tgHD rats compared to WT rats (WT: 7675.0±4972 vs HD: 1697±1139) (Fig. 1 A-C). As some of the proliferating cells in the SVZ are known to divide symmetrically and to maintain the stem cell pool, we analysed the number of cells that incorporated BrdU and remained as such in the SVZ 4 weeks after BrdU labelling (daily administration of BrdU on 5 consecutive days). The total number of BrdU label-retaining cells was significantly lower in the SVZ of tgHD rats compared to WT rats (WT: 670±330 vs HD: 170±80) (Fig. 1 D-F). Thus, the SVZ of tgHD rats has fewer proliferating stem cells compared to WT animals.

### Reduced cell proliferation in the OB of tgHD rats

**Fig 2:**
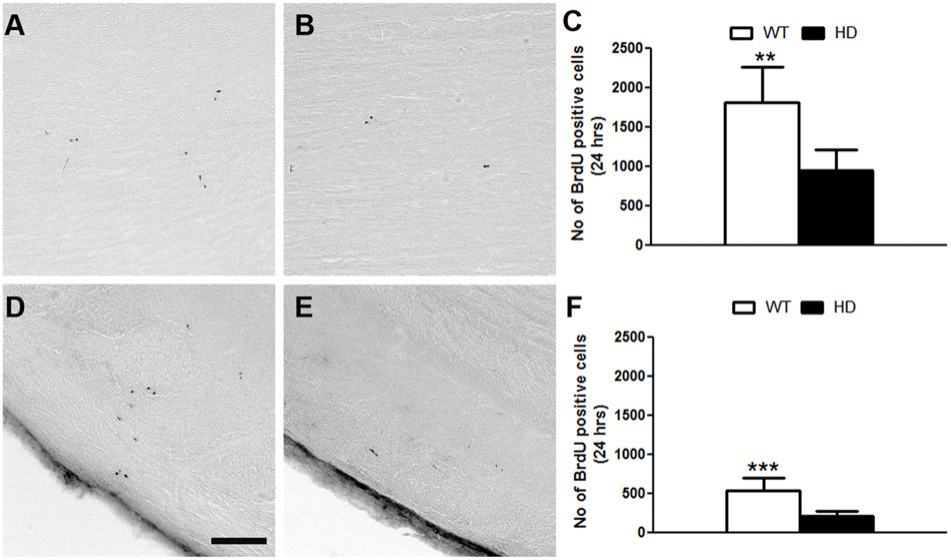
A, B) BrdU positive cells (24h) in the GCL and GLOM of A,D) WT and B,E) tgHD rats respectively. Quantitative analyses of BrdU positive cells after 24 hours treatment in the C) GCL and F) GLOM of the OB. Note, there is a significant reduction in proliferating cells in the GCL (WT = 4, HD = 8; Student t test, P value = 0.0020) and GLOM (WT = 4, HD = 8; Student t test, P value = 0.0006) of OB in tgHD rats compared to WT. Sale bar = 100μm.

For the OB, besides the well-described migration of neuroblasts from the SVZ through the rostral migratory stream, the resident progenitor cells have also been proposed as local source for new neurons [31,32]. Moreover, differential proliferative responses have been described in the post ischemic hippocampus, temporal cortex, and olfactory bulb of young adult macaques [33]. Thus, we asked whether, besides the reduced number of proliferating cells in the SVZ, there might be also fewer cells proliferating in the OB of tgHD rats. The quantitative analysis of BrdU^-^positive cells 24 h after BrdU labelling revealed a significant reduction in the cell proliferation in the tgHD GCL (WT: 1804±458 vs HD: 969±267) (Fig. 2 A-C) and in the GLOM (WT: 529±169 vs HD: 205±64) (Fig. 2 D-F) compared to the respective layers of the OB in WT. Thus, similar to the situation in the SVZ, the OB of tgHD rats contains fewer proliferating progenitor cells compared to the OB of WT animals. The mean volumes of SVZ and OB in the tgHD rats were not significantly different from the WT rats (data not shown).

### Increased dopaminergic neuronal fate in the OB of tgHD animals

**Fig 3:**
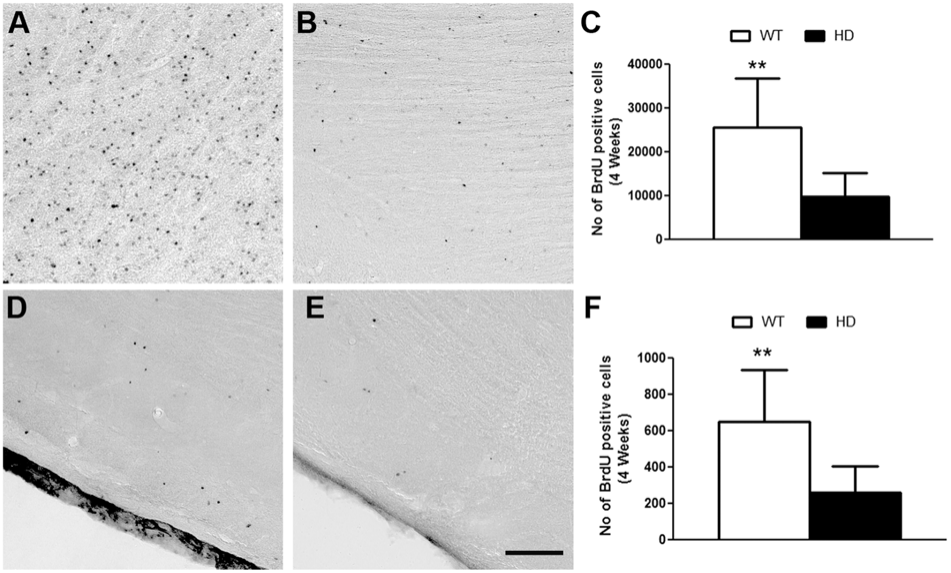
A, B, D, E) BrdU positive cells after 4 weeks in the GCL and GLOM of A, D) WT and B, E) tgHD rats respectively. Quantitative analyses of BrdU positive cells after 4 weeks in the C) GCL and F) GLOM of the OB. Note, there is a significant reduction in cell survival in the GCL (WT = 4, HD = 8; Student t test, P value = 0.0066) and GLOM (WT = 4, HD = 8; Student t test, P value = 0.0097) of OB in tgHD rats compared to WT. Sale bar is 100 μm.

We further investigated the fate of newly generated cells in the OB, i.e. of cells that incorporated BrdU, survived for 4 weeks in the OB. The analysis revealed that the total number of BrdU-positive cells was drastically reduced in the GCL (WT: 25605± 11086 vs HD: 9738± 5448) (Fig. 3 A-C) and in the GLOM (WT: 646 ± 285 vs HD: 258 ± 147) (Fig. 3 D-F) of tgHD rats compared to WT. The ratio between the cell proliferation in the SVZ and survival of cells in the OB was similar in the WT and in the tgHD animals. Therefore, the reduced number of BrdU-positive cells appeared to be derived from a reduced proliferation, but not from a reduced cell survival.

**Fig 4:**
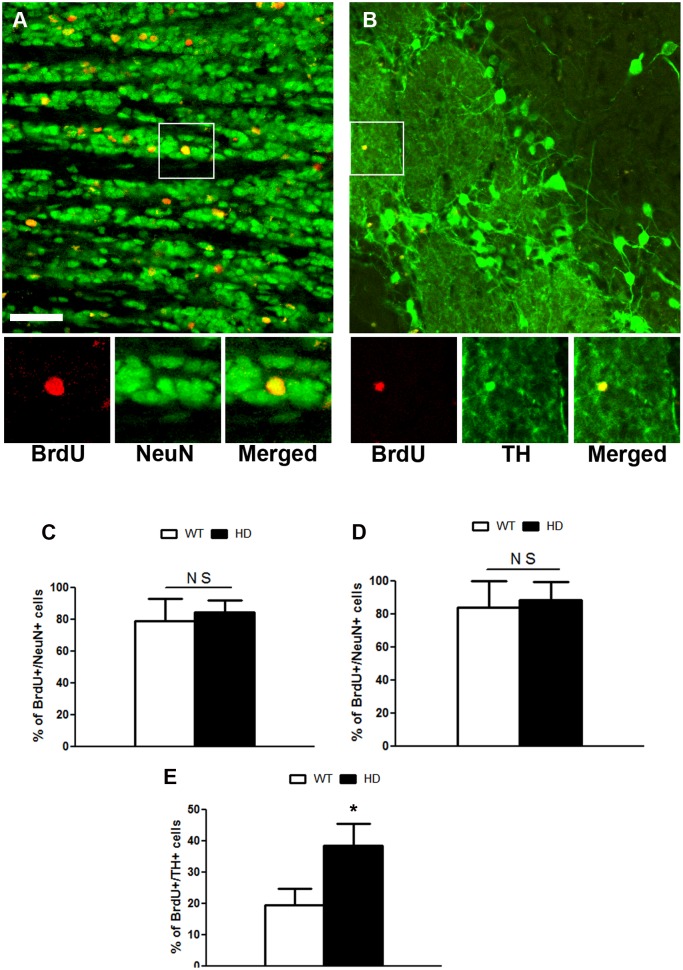
A) BrdU(red)/NeuN (green) positive in the GCL of OB in WT rats. Quantitative analysis of the percentage of BrdU/NeuN positive cells in the C) GCL and D) GLOM. Note, there is no change in the neuronal differentiation in the GCL (WT = 4, HD = 8; Student t test, P value = 0.3839) and GLOM (WT = 4, HD = 8; Student t test, P value = 0.5673) of OB between WT and tgHD rats respectively. B) BrdU(red)/TH(green) positive cells in the GLOM of OB in tgHD rats. E) Quantitative analysis of the percentage of BrdU/TH positive cells. Note, there is a significant increase in the percentage of BrdU/TH positive cells in the GLOM of OB of tgHD rats compared to WT rats(WT = 4, HD = 8; Student t test, P value = 0.0213). Insets are higher magnifications of the selected fields. Sale bar is 100 μm.

Next, we determined the neuronal phenotype of newly generated cells in the GCL and in the GLOM by confocal analysis of BrdU/NeuN double staining (Fig. 4 A-B). As a result, the percentage of BrdU/NeuN-positive cells was not altered in the GCL (WT: 79.0 ± 13.5 vs HD: 84.4 ± 7.4) (Fig. 4 C) and in the GLOM (WT: 84.0 ± 15.7 vs HD: 88.5 ± 10.9) (Fig. 4 D) of tgHD rats compared to WT rats. As a subpopulation of newly generated cells differentiates into dopaminergic neurons in the GLOM [26], we analyzed the percentage of BrdU/TH positive cells in this structure. We observed an increased percentage of BrdU/TH double-positive cells in the GLOM of tgHD rats (WT: 19.3±5.4 vs HD 38.3±7.1) compared to WT rats (Fig. 4 E) thereby suggesting either an induction or a selection of dopaminergic neurons in the GLOM of tgHD rats.

### Deviation of neuroblasts from the SVZ to the striatum in tgHD rats

**Fig 5:**
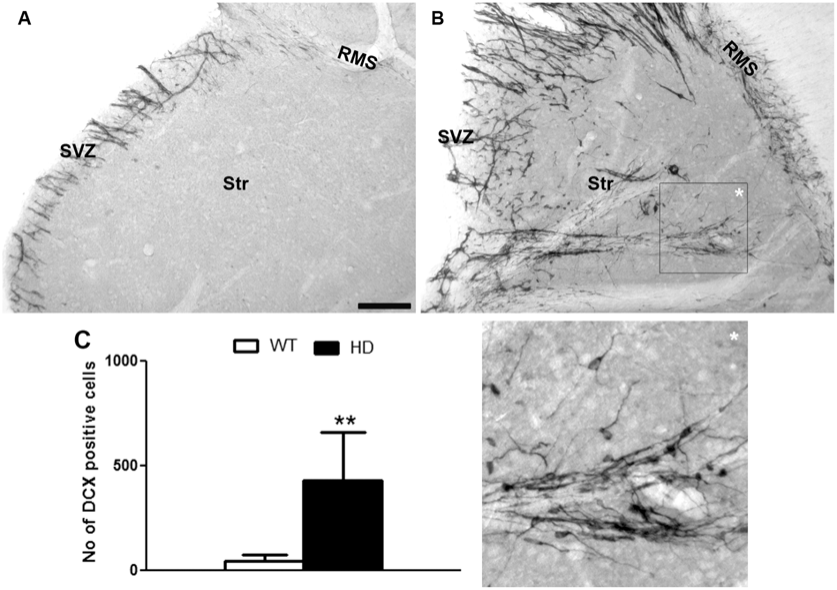
A, B) DCX positive cells in the striatum of A) WT and B) tgHD rats. C) Quantitative analyses of DCX positive cells in the striatum of the OB. Note, there is a notable appearance of DCX positive cells in the striatum of tgHD rats which resulted in a significant increase in DCX positive cells in striatum of tgHD rats compared to WT (WT = 4, HD = 8; Student t test, P value = 0.0084). *An inset is higher magnifications of the selected field. Sale bar is 100 μm.

Previously, we reported that DCX-positive neuroblasts migrate from the SVZ into the striatum in the R6/2 mouse model [26]. To investigate if this ectopic appearance of DCX-positive cells is specific to the R6/2 mouse model or if it might be a general feature associated with HD pathomechanisms, we analyzed the presence of DCX-positive cells in the striatum of tgHD rats. In the lateral ventricle wall/striatal region of WT animals, the presence of DCX-positive cells was largely confined to the SVZ-RMS system (Fig. 5A). Though few DCX-positive cells were found in the striatum adjacent to the SVZ and RMS as reported earlier [34], almost no DCX-positive cells were present in the innermost striatum of WT rats. In contrast, in the striatum of tgHD rats a dense population of DCX-positive cells appeared (Fig. 5B). Many of the DCX-positive cells were aligned in chains stretching from the SVZ deep into the striatum suggesting that these DCX-positive cells might indeed be derived from the SVZ (Fig. 5B). The quantitative analysis revealed that the total number of DCX-positive cells in the striatum of tgHD animals was ~10-fold higher compared to WT animals (WT: 43±32 vs HD: 430±230) (Fig. 5C). Thus, it seems that the SVZ of tgHD shows a robust neurogenic response to HD pathology thereby supplying immature neurons to the striatum, the most vulnerable region affected in the HD [17,26].

**Fig 6:**
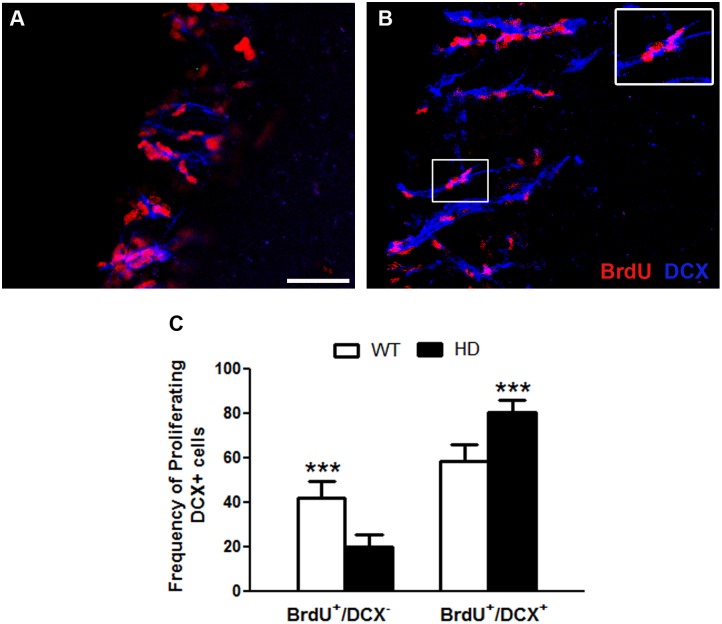
A, B) BrdU(red)/DCX(blue)positive in the SVZ of A) WT and B) tgHD rats. C,D) Quantitative analysis of the percentage of BrdU/DCX positive cells. Note, there is significant increase in the percentage of BrdU/DCX positive cells in the SVZ of tgHD rats compared to WT rats (WT = 4, HD = 8; One way ANOVA and Tukey’s Comparison Test, P value = P<0.0001). An inset is higher magnifications of the selected field. Scale bars = 50 μm.

In our previous study, which focused on the hippocampal neurogenic niche in the tgHD rat, we noticed that while fewer neural stem cells (NSCs) were actively dividing, a higher proportion of DCX-positive cells were still proliferating [29]. Therefore, we asked if the ectopic appearance of DCX-positive cells in the striatum of tgHD animals was due to an expansion of the pool of DCX-positive cells. Indeed, quantitative analysis of the animals sacrificed 24h after BrdU administration revealed that, while the reduction in proliferation in the tgHD SVZ was largely due to the DCX-negative population (lower number of BrdU^+^/DCX^-^), (WT: 41.6±7.6 vs HD: 19.6±5.5), the DCX-positive and proliferating population was significantly enlarged in these HD rats (WT: 58.3±7.6 vs HD: 80.3±5.5) (Fig. 6 A-C). Interestingly, incorporation of BrdU was detected in DCX-positive cells that were specifically located in the SVZ adjacent to the striatum of tgHD rats (Fig. 6 B). Surprisingly, we were not able to find DCX/BrdU-double positive cells in the innermost area of the striatum of tgHD rats. This suggests that the DCX-positive cells found in the striatum of tgHD rats are recruited by a proliferating pool of DCX-positive cells present in or in proximity to the SVZ.

**Fig 7:**
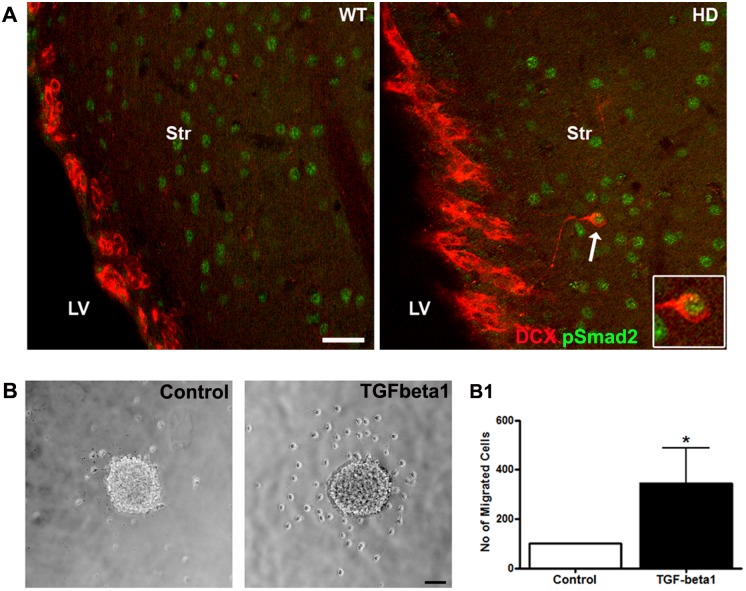
A) TGF-beta signaling in DCX positive cells. DCX (red) positive cells in the striatum but not in the SVZ co-label with pSmad2 (green). Scale bars = 50 μm. B) Different migration potential of neuropheres treated with TGF-beta1 or with vehicle; C) quantitative analysis of number of cells that migrated out of the neurospheres (Control = 3, TGFbeta1 = 3; Student t test, P value = 0.0483). Scale bars = 100 μm.

Further, we investigated on the molecular mechanisms, in particular on the extracellular cytokines, which might trigger the migration of the DCX-positive cells into the striatum. Based on our previous data on TGF-beta-Smad2 pathway [29,35,36] and on recent report using a Cre-LoxP based conditional model for ALK5 [37], we hypothesized that TGF-beta1 signaling might be involved in the migration phenotype of striatal DCX-positive cells and used phospho (p)-Smad2 immunostainings as a readout of active TGF-beta1 signaling [29,36]. Indeed, DCX-positive cells in the striatum were co-labelled with pSmad2 (Fig. 7 A) suggesting active TGF-beta signaling in these cells. Further, we used a migration assay of adult WT rat SVZ derived neurospheres [30,38] to validate the effects of TGF-beta1 on the migrational potentials of neuronal precursor in vitro (Fig. 7 B). TGF-beta1 or vehicle treated cells were seeded on poly-L-ornithine and laminin-coated coverslips and 18 h later the number of cells that migrated out per neurosphere was determined. Significantly more cells migrated out from the TGF-beta1 treated neurospheres as compared to the vehicle conditions (Fig. 7 B).

**Fig 8:**
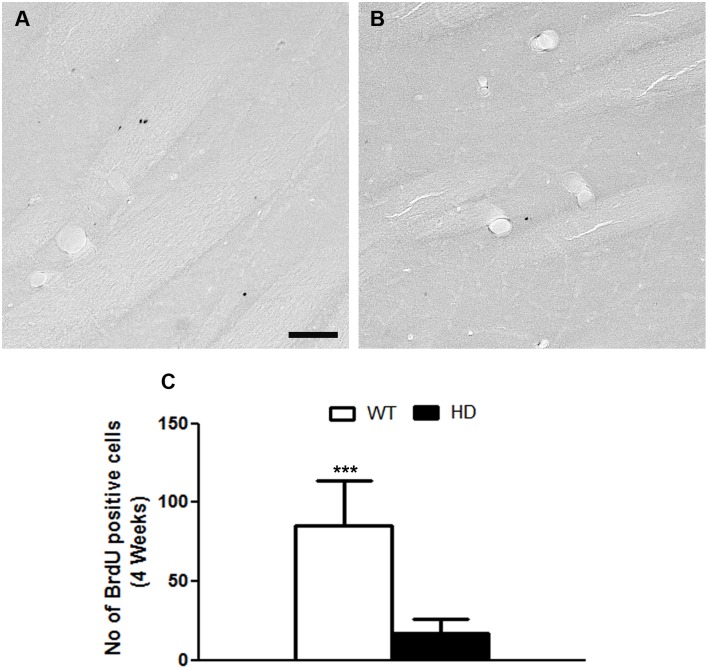
A, B) BrdU positive cells after 4 weeks in the straitum of E) WT and F) tgHD rats. C) Quantitative analysis of BrdU positive cells after 4 weeks treatment. Note there is a significant reduction in cell survival in the SVZ of tgHD rats compared to WT (WT = 4, HD = 8; Student t test, P value = 0.0001). Scale bar = 100 μm.

To analyze the fate of newly generated cells that had either originated in or migrated to the striatum of tgHD rats, we analyzed cells that had incorporated BrdU and survived 4 weeks after BrdU labelling. We noticed that the total number of BrdU-positive cells was significantly lower in the striatum of tgHD rats compared to WT rats (WT: 85.2±28 vs HD: 17.14±8.7) (Fig. 8 A-C). The BrdU-positive cells were always negative for DCX or NeuN regardless of the genotype of the animals indicating that these cells did not terminally differentiate into neurons.

## Discussion

In the present study, we demonstrate that cell proliferation in the SVZ of a tgHD rat model is reduced compared to age-matched WT controls. This observation is in contrast with previous proliferation data observed in the SVZ of other rodent models, where either unchanged or increased proliferation rates were noted [23,26], and SEL of human HD brains, in which an increased proliferation of neuronal and glial was reported [22]. Obviously, differences in species, age, neuropathological status, and a different identity of the proliferating cells might account for such discrepancies. In a previous study, we had investigated and compared 8 month and 12 months old animals of the same tgHD rat model, which we used in the present study, and we observed a reduced cell proliferation in the hippocampus dentate gyrus of 12 months old but not of 8 months old tgHD rats [29] supporting the hypothesis of age- or neuropathology status-dependant effects on cell proliferation in the neurogenic niches. The reason for the inconsistency of proliferation data in the SVZ regions between human and different genetically modified animal models of HD is unclear. One possible explanation could be the difference in the life span and / or the extent of neuropathology present in the brains of rodents and humans, and even between the different HD animal models. For example, the herein used tgHD rats have a life expectancy similar to WT rats [28], while the transgenic mouse models (R6/1 or R6/2) have a much shorter life span compared to the WT counterparts [27]. Of course, there are dissimilar pathological grades in brains of different experimentally induced HD models as they express varying lengths of CAG repeats of human HTT and display differences in disease phenotype [4,27,28]. Nevertheless, the neurodegeneration and cell death in the striatum of tgHD rats has been extensively characterized, which supports the clinical relevance of this animal model [39].

The reduced number of cells labelled with BrdU 4 weeks after BrdU injection and found in the SVZ of tgHD rats could be caused by various factors like reduced proliferation, reduced self-renewal, prolonged cell cycle, and increased death of NPCs. Interestingly, we noticed an increased number of proliferating DCX positive neuroblasts followed by their migration towards the striatum of tgHD rats, similar to the situation of an increased number of proliferating DCX positive cells in the hippocampus of the tgHD rats [29]. The induced mitotic activity of precursor cells, in the present case of robust number of proliferating DCX positive cells, might result in depletion of NPCs [29,40]. Therefore, the selective expansion of DCX positive neuroblasts (Fig. 6) might be crucially contributing to the reduced levels of BrdU label-retaining cells in the SVZ of tgHD rats.

Increased cell proliferation in the SVZ and ectopic migration of neuroblasts have been observed in the striatum of MCAO-induced ischemic stroke models [41,42,43,44], and also after brain hemorrhage caused by injection of bacterial collagenase or blood [45,46,47], as well as in acute models of Parkinson’s Disease (PD) after administration of epidermal growth factor (EGF) and fibroblast growth factor 2 (FGF2) [48], and, moreover, after application of Noggin and brain-derived neurotrophic factor (BDNF) in adult rats [49]. In some cases, ectopically migrated neuroblasts even differentiated into medium spiny neurons (MSNs) in the acutely damaged striatum, but in most cases they failed to survive [50]. Recently, we and others have shown a re-direction of neuroblasts from the SVZ towards the striatum of R6/2 mice [26,51]. In the present study, we found an even more drastic migration pattern of DCX positive neuroblasts from the SVZ to the striatum of tgHD rats, thereby confirming our previous findings in R6/2 mice [26]. At present, we would like to propose a novel term “reactive neuroblastosis” to indicate the selective induction of DCX positive cells to proliferate and to invade the non-neurogenic region in degenerated adult brains based on the following evidences: i) the cells emerge specifically in response to a neuropathology, therefore considered as “reactive”, ii) they are mitotically active, and iii,) they show young immature neuronal features, including the migration pattern, therefore considered as neuroblasts. There has been increasing amount of data demonstrating the evidences of proliferating DCX positive cells in non-neurogenic regions in response to acute neuronal damage or progressive neurodegeneration. Thus this feature might not be restricted to a particular situation but it can be a general course of counter action against many different neuropathologies or response to naturally or experimentally induced neurogenic stimuli for some extent. For example, proliferating DCX cells appear in clusters in a transgenic mouse model of progressive striatal degeneration [52]. Also, in response to stroke, DCX positive cells deviate from the SVZ to the striatum, where they keep on dividing [53]. Moreover, actively dividing DCX positive cells have been demonstrated in post-mortem brain specimen of patients with mesial temporal lobe epilepsy [54]. It further needs to be addressed, whether an induction of neural stem cells or astrocytes or other cell types are contributing to the genesis of the surplus neuroblasts. It can be assumed that the ectopically migrating DCX positive cells may differ in their identity and functional properties from stream-associated DCX positve cells. Therefore, 1) the origin and the molecular and cellular basis of reactive neuroblasts in non-neurogenic regions, 2) their beneficial or detrimental effects, and 3) identifying substrates that elicit neuroblasts are the important aspects of further investigation in many neuropathological situations.

The most widely recognized mechanism regulating the migration of neuroblasts is SDF1α signalling through its receptor CXCR4 [55,56,57]. However, the mechanisms regulating neuroblast migration in the striatum of HD is not yet identified. We have recently described that elevated levels of TGF-beta1 signalling in the hippocampus of tgHD rats and R6/2 mice [29,35]. Besides, it is well documented that TGF-beta1 could alter the expression of many different integrins, cell adhesion molecules, extracellular matrix and other factors important for cellular migration and, in addition, exerts growth-inhibitory responses in normal cells, but induces migration and invasion of neoplasms [58]. Also, TGF-beta1 blocks proliferation of neural progenitor cells and promotes stem cell quiescence and neuronal survival / differentiation [36,59]. Our present study supports the hypothesis that TGF-beta1 might be a crucial factor regulating the migration of DCX positive neuroblast, as we have noticed the appearance of pSmad2 in the nucleus of DCX positive neuroblasts in the RMS of WT rats and also in the striatum of tgHD rat. However, DCX positive neuroblasts that are found in the striatum of tgHD rats mostly failed to survive, which, nevertheless, could be explained by an adverse microenvironment that is presumably caused by the mutant Htt protein in the striatum of tgHD rats as previously hypothesised [26]. Another factor that could contribute to the failure of survival of striatal neuroblasts could be a prolonged activation of inflammatory cells such as activated microglia and resulting circulation of pro-inflammatory molecules in the diseased brain, which can be detrimental to terminal neuronal differentiation, integration and survival [60,61,62].

Dysregulation of CREB signaling is linked to neuronal loss in HD [29,63]. Recently Luzzati and colleagues [52] demonstrated that the migration of SVZ derived neuroblasts to the striatum in the Creb1^Camkcre4^/Crem^-/-^ mutant mice (CBCM) is due to the neurodegeneration in the striatum. Proliferation was not altered in the SVZ of the CBCM model that corroborates our previous findings in R6/2 mouse model [26]. The individual neuroblasts that were found in the striatum of CBCM have been shown to originate from the SVZ. Actively proliferating DCX clusters were suggested to arise from the striatal specific NPCs. In contrast, we observed that proliferation was reduced in the SVZ of tgHD rats, but proliferating DCX cells were in the striatum in proximity to the SVZ. Although these cells appear to have migrated from the SVZ, as they show the typical chain migration pattern, they did not show any mitotic activity in the inner most striatum of tgHD rats. Therefore, the pathophysiology of the CBCM mice model appears to be different from tgHD rats.

Our recent data on the forebrain of R6/2 mice revealed that, while the mutant Htt protein, at least in its aggregate form was expressed in DARPP-32 positive mature GABAergic neurons in the striatum, it was not expressed in migrating DCX positive neuroblasts [26]. Therefore, the detrimental effects of the mutant Htt protein might be a limiting factor for maturation and survival of newly migrated immature neurons in the striatum of tgHD rats. We hypothesize that the onset of Htt expression could coincide with cell death of striatal neuroblasts in the tgHD rats. Thus, the enhanced migration of neuroblasts observed in the HD striatum may be a failed attempt for compensatory neurogenesis. In order to develop therapeutic strategies to enhance neuronal integration and survival in HD brain it is important to decipher mechanisms that are involved in the fate of newly migrated neuroblasts to the striatum of HD brains. Further studies, using reporter-based *in vivo* surveillance [64,65] on neuroblasts may provide more details of spatiotemporal origin, migratory patterns and fate of migrating neuroblasts in the striatum of pre-clinical animal models.

A number of CNS disorders have been shown to be apparently associated with altered olfactory function including schizophrenia [66,67], PD [68] and HD [69]. While the occurrence of neurogenesis needs to be convincingly explored in the OB of human adult brain [70,71,72], in the rodent reduced neurogenesis in the OB was correlated with dysfunction of olfactory discrimination [73]. In contrast, the increased survival of newly born neurons in the OB was associated with improved olfactory-dependent memory [74,75]. Our previous observation in R6/2 mice demonstrated reduced neuronal survival in the GCL and GLOM of the OB [26]. In the present study, we confirmed a significant reduction of proliferation in the GCL and GLOM of tgHD rats. One explanation for reduced proliferation observed in the OB could be the reduced proliferation of NPCs in the SVZ of tgHD rat, which in turn leads to a reduced migration of neuroblasts along the RMS. The reduced cell survival in the OB of tgHD rats might be the result of reduced proliferation, the impact of ectopic migration of neuroblasts to the striatum and impaired neuronal integration. Based on the increased TUNEL staining in the OB of R6/2 mice in our earlier studies we hypothesised that increased cell death might play a major role in decreased cell survival [26]. Therefore, an increased cell death together with effects of the mutant Htt protein might culminate in reduced cell survival in the OB of tgHD rats. In the OB of the adult brain, the GCL is populated mainly by GABAergic neurons including different subpopulations of inhibitory interneurons [76,77]. In contrast to decreased neuronal differentiation in the hippocampus [29], the frequency of neuronal differentiation in the OB of tgHD rats was not changed regardless to the neurotransmitter type. Thus, there might be an intrinsic difference between hippocampus-derived NPCs and SVZ-derived NPCs [78]. Further, the maturation of neuroblasts that are derived from the SVZ is normally modulated by the microenvironment of the OB, and this might explain the niche specific difference between OB and hippocampus. Interestingly, tgHD rats displayed an increased differentiation frequency of TH positive dopaminergic neurons in the GLOM. Previously, the increased frequency of dopaminergic lineage in the OB has been reported in an animal model of Parkinsonism [79]. In this PD model, the nigrostriatal lesion was induced by injecting 6 hydroxydopamine (6-OHDA) into the striatum. This alteration in the nigrostriatal dopamine system has been proposed for the increased frequency of dopaminergic differentiation in the OB [79]. Besides, the preferential differentiation shift of NPCs to the dopaminergic phenotype in the OB of tgHD brains might be an endogenous escape mechanism, as the neurodegeneration resulting from mutant Htt protein may not act on dopaminergic neurons. Further studies are needed to understand the differentiation fate of NPCs with regards to the neurotransmitter phenotype in the normal and HD brains.

**Fig 9:**
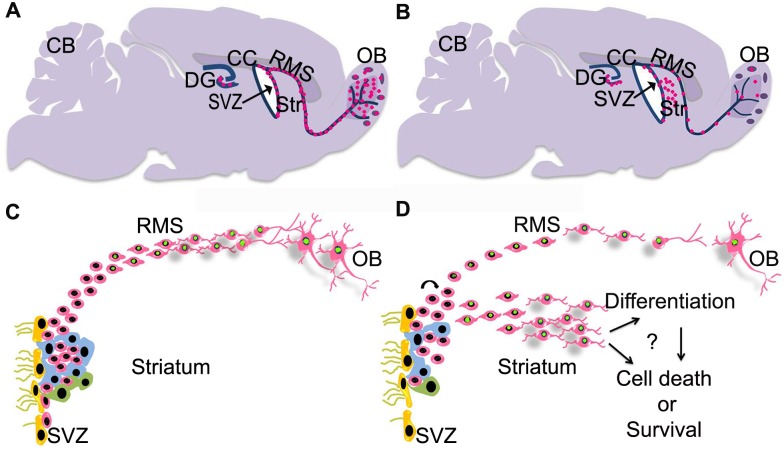
A-B) An illustration of neurogenesis in the SVZ-RMS-OB A) WT vs B) tgHD rats. C-D) Schematic representation of migration of precursors cell along the RMS-OB of C) WT rats and D) ectopic migration of neuroblast and appearance of pSmad2 signal (Green) in the striatum and reduced neurogenesis in the OB of tgHD. CB; Cerebellum, Ctx;cortex, DG; dentate gyrus, SVZ; Subventricular zone, Stri; Striatum, RMS; Rostral Migratory Stream, OB; Olfactory Bulb.

In summary, the present study demonstrates that the proliferation of NPCs is reduced in the SVZ of tgHD rats compared to WT rats. In contrast, DCX positive cells respond with a clear hyperproliferation and with an extensive migration into the striatum. Even though, we observed a selective proliferation of neuroblasts in the areas of the striatum adjacent to the SVZ of tgHD rats, the innermost striatum of tgHD rat showed no evidence for the neuronal maturation and survival (Fig. 9).
